# A Study Based on b-Value and Information Entropy in the 2008 Wenchuan 8.0 Earthquake

**DOI:** 10.3390/e27040431

**Published:** 2025-04-16

**Authors:** Shasha Liang, Ziqi Wang, Xinyue Wang

**Affiliations:** 1 Inner Mongolia Regional Seismological Bureau, Hohhot 010010, China; 2 School of Mathematical Sciences, Applied Statistics, Inner Mongolia University South Campus, Hohhot 010021, China

**Keywords:** micro rupture, acoustic emission b-value, information entropy

## Abstract

Earthquakes, as serious natural disasters, have greatly harmed human beings. In recent years, the combination of acoustic emission technology and information entropy has shown good prospects in earthquake prediction. In this paper, we study the application of acoustic emission b-values and information entropy in earthquake prediction in China and analyze their changing characteristics and roles. The acoustic emission b-value is based on the Gutenberg–Richter law, which quantifies the relationship between magnitude and occurrence frequency. Lower b-values are usually associated with higher earthquake risks. Meanwhile, information entropy is used to quantify the uncertainty of the system, which can reflect the distribution characteristics of seismic events and their dynamic changes. In this study, acoustic emission data from several stations around the 2008 Wenchuan 8.0 earthquake are selected for analysis. By calculating the acoustic emission b-value and information entropy, the following is found: (1) Both the b-value and information entropy show obvious changes before the main earthquake: during the seismic phase, the acoustic emission b-value decreases significantly, and the information entropy also shows obvious decreasing entropy changes. The b-values of stations AXI and DFU continue to decrease in the 40 days before the earthquake, while the b-values of stations JYA and JMG begin to decrease significantly in the 17 days or so before the earthquake. The information entropy changes in the JJS and YZP stations are relatively obvious, especially for the YZP station, which shows stronger aggregation characteristics of seismic activity. This phenomenon indicates that the regional underground structure is in an extremely unstable state. (2) The stress evolution process of the rock mass is divided into three stages: in the first stage, the rock mass enters a sub-stabilized state about 40 days before the main earthquake; in the second stage, the rupture of the cracks changes from a disordered state to an ordered state, which occurs about 10 days before the earthquake; and in the third stage, the impending destabilization of the entire subsurface structure is predicted, which occurs in a short period before the earthquake. In summary, the combined analysis of the acoustic emission b-value and information entropy provides a novel dual-parameter synergy framework for earthquake monitoring and early warning, enhancing precursor recognition through the coupling of stress evolution and system disorder dynamics.

## 1. Introduction

Current earthquake prediction predominantly relies on empirical methods that lack physical interpretability. The integration of multi-parameter indicators reflecting different mechanical processes may bridge this gap. This reality forces earthquake prediction research to break through the observation blind spots of traditional empirical methods [1] and instead establish a dynamic monitoring system based on physical evolution mechanisms. In this context, the acoustic emission b-value and information entropy, as dual indicators with strong physical interpretability, provide complementary research perspectives: the former reveals the evolution trend of a regional stress field through the statistical essence of the magnitude–frequency relationship [2,3], while the latter captures the jump into nonequilibrium of the spatiotemporal structure of seismic activities from the perspective of the entropy increase principle [4,5]. In theory, coupling the analysis of the two can build a bridge between the microscopic fracture mechanism and macroscopic system instability, but the construction of their collaborative path is still restricted by the theoretical absence and practical bottlenecks of multi-parameter fusion. This study innovatively combines the dual-indicator method of the acoustic emission b-value and information entropy, applying it to the 2008 Wenchuan earthquake sequence to decode precursor characteristics during the stress accumulation phase.

The inherent flaws of single-parameter analysis methods show a tendency to become obvious in earthquake precursor detection. For the acoustic emission b-value, its physical essence is rooted in the homogeneity assumption of the mechanical properties of rock media, while the multi-scale fracture network in natural fault zones will inevitably lead to the spatial heterogeneity of parameter responses [3,6]; the dynamic adjustment of the regional tectonic stress field further makes the single-threshold criterion lose its universality when crossing plate boundaries [2]. The information entropy method is limited by the open characteristics of the dynamic system—the observational errors of the micro-earthquake catalog and the aliasing of non-tectonic interference events will distort the true trajectory of the evolution of the entropy value, resulting in misjudgments in the “pseudo-ordering” of precursor signals [7,8]. The existing synergy models attempt to simply linearly superimpose the two, but ignore the multi-level coupling mechanism of earthquake precursor generation: there is a nonlinear interaction between the energy dissipation characteristics of b-value changes and the spatiotemporal correlation of information entropy, and the fixed-weight model is difficult to adapt to the weight differences of the dominant physical processes in different tectonic environments [9,10]. This theoretical gap directly leads to the fact that the precursor identification efficiency of existing methods in complex tectonic areas such as the eastern margin of the Qinghai–Tibet Plateau is significantly weaker than that at the stable plate edges [3,5].

This study takes the Wenchuan earthquake as a sample, conducting systematic experimental observations and theoretical analyses to deeply explore the cross-scale dynamic similarities between rock instability experiments and natural earthquakes. The 2008 Wenchuan 8.0-magnitude earthquake occurred at 14:28 Beijing Time (06:28 Coordinated Universal Time) on May 12, with its epicenter located at 31.0° N, 103.4° E, within the Longmen Shan Fault Zone in Sichuan Province, China. This fault zone serves as a tectonic boundary between the Tibetan Plateau and the Sichuan Basin and exhibits high seismic activity due to the ongoing collision between the Indian and Eurasian plates. The earthquake resulted in a surface rupture extending 270 km, making it a typical case of intracontinental thrust faulting. The research findings indicate that the b-value descending phase transition and the entropy convergence state share a significant homology in their physical mechanisms, which enhances our understanding of the earthquake gestation process. Moreover, the conclusions drawn from this study can provide valuable references for the design of earthquake early warning systems in other regions.

## 2. Principles and Methods

### 2.1. Acoustic Emission b-Value

An acoustic emission (AE) is a high-frequency acoustic wave that is released when a material under stress experiences microscopic damage. The b-value of AE is an important parameter that characterizes the energy distribution of an AE event and is used to quantify the damage activity of a material during the loading process. B-value variations can reflect the microscopic damage mechanism of a material, which can provide important information for seismic prediction.

In seismology, the b-value is usually related to the magnitude distribution of seismic activity, described in particular by the Gutenberg–Richter relationship (also known as the G-R relationship). This relationship is a statistical model for representing the relationship between the magnitude of an earthquake and the number of earthquakes that occur in a given area and time frame. Its formula is

(1) $$\log_{10}N=a-bM$$

where M is the magnitude, N is the number of earthquakes with a magnitude greater than M occurring in a certain area within a certain time range, parameter a is related to the intensity of background seismicity in the study area, and parameter b describes the proportion of large and small earthquakes in the study area, which is related to the magnitude distribution of earthquakes, and it reflects the frequency of earthquakes of larger magnitude, which is what we call the b-value. There are two ways to calculate the acoustic emission b-value, i.e., the least squares method and the maximum likelihood estimation method [6]. In this paper, we use the maximum likelihood estimation method to calculate the acoustic emission b-value; the calculation method is as follows:

According to the likelihood principle, i.e., choosing a model expression that seeks to maximize the probability of generating model parameter values for the observed data, we have to find the appropriate values of a and b such that the probability of occurrence of the magnitude data of the actual recorded seismic event is maximized for a given value of a and b, i.e., Mi.

So by the G-R law, we have

(2) $$N=10^{a-bM}$$

If the starting magnitude (amplitude threshold) is Mc, the relevant expression for the distribution function F(M) (i.e., denoting the probability that the random variable takes on a value less than or equal to M) based on the magnitude event is

(3) $$p\left(Mag\gg M\right)=1-F\left(M\right)=\frac{N\left(M\right)}{N\left(M_{c}\right)}=10^{-b\left(M-M_{c}\right)}=e^{-\beta (M-M_{c})}$$

Among others, β=bln⁡10≈2.3b.

Then the probability density function f(M) (which describes the likelihood of a random variable in the vicinity of some definite value point) is

(4) $$f\left(M\right)={\left[F\left(M\right)\right]}^{\prime }=\beta e^{-\beta (M-M_{c})}$$

So, in a set of observations (Mi, I = 1, 2, 3, …, N), N is the total number of events that satisfy M ≥ Mc. Therefore, there is a likelihood function:

(5) $$L=\prod_{i=1}^{N}f(M_{i})=\beta^{N}e^{-\beta \sum_{i=1}^{N}\left(M-M_{c}\right)}$$

To maximize the likelihood function, i.e., to maximize the value of Y equal to ln L, let

(6) $$\frac{\partial Y}{\partial \beta }=0$$

Substitutions can be made:

(7) $$\hat{\beta }={\left(\bar{M}-M_{c}\right)}^{-1}$$

Assume (office)

(8) $$\hat{b}=\frac{\log_{10}e}{\bar{M}-M_{c}}\approx \frac{0.4343}{\bar{M}-M_{c}}$$

The physical significance of the b-value is that higher b-values (usually greater than 1) imply a more uniform energy distribution of the acoustic emission event, suggesting that the material is relatively stable at the microscopic level and that the damage is dispersed, whereas lower b-values (less than 1) are indicative of a more concentrated damage activity that may signal a potential risk of seismicity. A lower b-value is usually associated with the concentration and release of pre-earthquake stress, providing the possibility of recognizing earthquake precursors.

In earthquake prediction, dynamic monitoring of the acoustic emission b-value can identify potential seismic activity: when there is a significant decrease in the b-value, it may imply that the accumulation of stresses within the crust has reached a critical point, thus increasing the likelihood of an impending earthquake. Therefore, analyzing the acoustic emission b-value can provide a quantitative basis for earthquake prediction.

In summary, the acoustic emission b-value has important theoretical and practical significance in the field of material science and earthquake prediction, and its variation not only reflects the microscopic damage mechanism of materials but also provides a reliable quantitative index for the identification of earthquake precursors.

### 2.2. Information Entropy

Information entropy (IE) is an important metric used to quantify system uncertainty and information content. It was first proposed by Claude Shannon [11] and used in information theory to describe the efficiency and reliability of information transmission. In the field of earthquake prediction, information entropy can be used to analyze the distribution characteristics of and dynamic changes in seismic events and reveal their potential regularities.

The process of calculating information entropy is as follows:

First, define the event and probability; let X be a discrete random variable with possible values X1, X2, …, Xn, and each event Xi occurs with a probability P(X_i_) that satisfies

(9) $$\sum_{i=1}^{n}P\left(x_{i}\right)=1$$

The information content of individual events is then calculated, which in information theory is a measure of the amount of information carried by a particular event when it occurs. The amount of information I(X_i_) for each event X_i_ is calculated as

(10) $$I\left(X_{i}\right)=-\log_{b}P(X_{i})$$

Here, b is the base of the logarithm. The common base numbers are

b = In bits (bit) at 2;
b = e in nanograms (nat).

As the probability of an event P(X_i_) is smaller, the more information it carries, I(X_i_). This is a way to quantify the amount of information carried by a small probability event.

The information entropy H(X) measures the uncertainty of the entire random variable X. We wish to compute the expected value of the amount of information for all possible events. To do this, we multiply the information content of each event by its probability and then sum again the overall event probabilities.

Therefore, the overall information entropy H(X) is calculated as

(11) $$H\left(X\right)=\sum_{i=1}^{n}P\left(X_{i}\right)(-\log_{b}P(X_{i}))$$

To wit,

(12) $$H\left(X\right)=-\sum_{i=1}^{n}P(X_{I})\log_{b}P(X_{i})$$

The significance of this formula is that it quantifies the uncertainty of all possible values of the random variable X in the form of entropy. A higher value of entropy indicates a higher level of uncertainty or instability in the system and vice versa [8].

If the probability of an event is 1, and the probability of the other events is 0, then the entropy H(X) = 0 indicates complete certainty.

The entropy is maximized if all events have an equal probability, calculated as

(13) $$H\left(X\right)=-n\left(\frac{1}{n}\log_{b}\frac{1}{n}\right)=\log_{b}n$$

With the above steps, we arrive at the formula for information entropy:

(14) $$H\left(x\right)=-\sum_{i=1}^{n}p(x_{i})\log_{b}P(X_{i})$$

where H(X) denotes the information entropy of the random variable X and represents the uncertainty of the random variable X. n is the total number of all possible events.

In earthquake prediction studies, information entropy is used to analyze the aggregation and regularity of seismic activity [11]. It has been shown that before an earthquake occurs, the crustal stress gradually concentrates in a certain region, resulting in frequent small seismic activities in that region but with a reduced overall randomness. This concentration of stress causes neighboring seismic events to exhibit more significant aggregation characteristics in time and space.

Earthquakes are often preceded by a series of small-scale foreshocks, and the occurrence of these foreshocks may form certain temporal and spatial patterns that could lead to a decrease in information entropy. When these foreshock activities are concentrated in a specific region, the system’s uncertainty may decrease, potentially resulting in a reduction in information entropy. At the same time, as the system approaches the critical point of earthquake occurrence, the motion and stress state of the Earth’s crust may become more organized, which could also lead to a decrease in information entropy. However, it is important to note that while these changes provide valuable insights into the physical processes of earthquake preparation, they do not guarantee the predictability of earthquakes.

In summary, information entropy has shown potential as a tool for analyzing seismic activity and identifying precursory signals. While its changes can provide important information about the state of the Earth’s crust, further research is needed to better understand its applicability and limitations in earthquake prediction. The study of information entropy could contribute to improving the theoretical understanding of seismic processes and providing additional data support for earthquake forecasting efforts. However, it is crucial to acknowledge that earthquake prediction remains a complex and challenging field, and no single method or indicator can ensure accurate and reliable predictions.

### 2.3. Combination of Acoustic Emission b-Value and Information Entropy

The acoustic emission b-value and information entropy, as two important metrics in earthquake prediction, each provide a unique perspective on seismic activity. Using the two together can effectively enhance the ability to recognize earthquake precursors.

The acoustic emission b-value reflects the degree of micro-damage of materials under stress, and its variation can reveal the accumulation and release of stresses within the crust, with lower b-values usually implying a concentration of stresses in the crust and an increased likelihood of earthquakes. Meanwhile, the information entropy quantifies the distribution characteristics and uncertainty of seismic events, which may further increase the likelihood of earthquakes.

Combining the study of the acoustic emission b-value and information entropy can help analyze dynamic changes in seismic activity more comprehensively. When both the b-value and information entropy show a decreasing pattern of change, it indicates that the crustal stress is in the accumulation stage, and the rupture of the rock body changes from a disordered state to an orderly state, which predicts that the risk of earthquakes is increasing. This multidimensional analysis method can provide a more reliable basis for earthquake prediction.

## 3. Data Processing and Analysis of Results

The data used in the data processing are continuous seismic waveform data recorded at six fixed stations around the Wenchuan 8.0-magnitude earthquake, which are sourced from seismic monitoring stations for statistical analysis and trend comparison. The data utilized in this study span from 1 April 2008 to 12 May 2008, encompassing 5243 seismic events (magnitude ≥ 1.5) recorded by six fixed stations (JYA, JMG, AXI, DFU, JJS, YZP). The data underwent band-pass filtering between 0.1 and 5 Hz, and events with a signal-to-noise ratio < 3 were excluded. All data were cleaned and preprocessed to ensure their accuracy and reliability, providing a solid database for the subsequent modeling and analysis.

### 3.1. Earthquake Faults and Station Distribution

**Figure 1:**
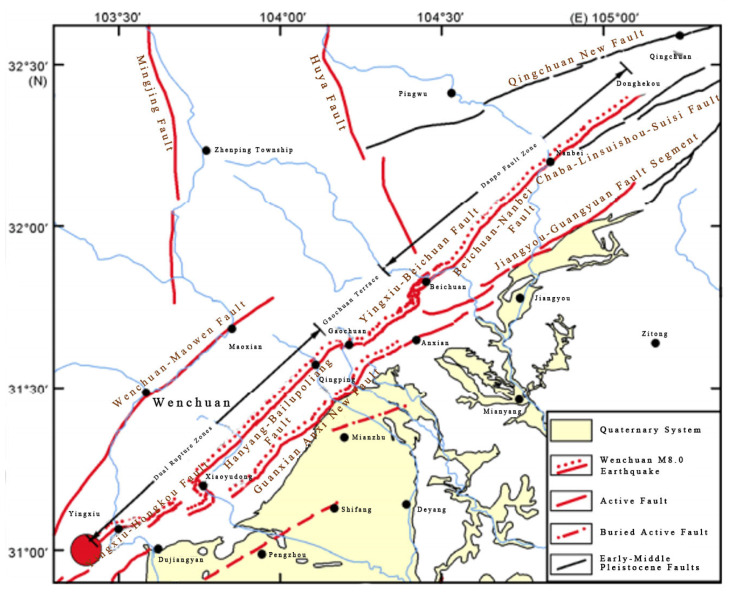
Distribution of surface rupture zones of the Wenchuan 8.0 earthquake [12].

**Figure 2:**
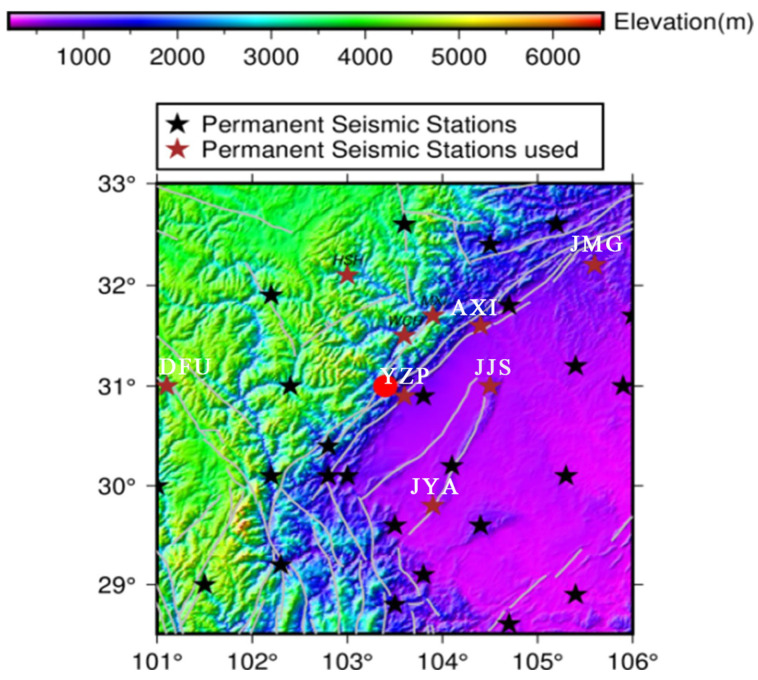
Distribution of fixed stations near the epicenter of the Wenchuan 8.0 earthquake (the red pentagrams are the six stations tracked by MASE).

The Wenchuan 8.0-magnitude earthquake is one of the major earthquakes that has occurred onshore in recent years, which had a profound impact on the geology and tectonics of the epicenter area. In order to better understand the geologic context of the earthquake and the distribution of stations near the epicenter, this study first shows the locations of the major faults in the earthquake area and the distribution maps of each fixed station used for data acquisition (see Figure 1 and Figure 2). These maps provide an intuitive understanding of the fracture structure and station locations, laying the foundation for the subsequent seismic signal analysis.

Figure 1 [12] shows the distribution of surface rupture zones and the location of the Longmenshan Fracture Zone in the Wenchuan 8.0-magnitude earthquake. The Longmenshan Fracture Zone is one of the major tectonic boundaries along the eastern margin of the Tibetan Plateau, characterized by a significant retrograde rupture, forming a complex rupture system. The fault zone consists of three major faults: the Wenchuan–Maoxian fault (also called the post-Longmenshan fault), the Yingxiu–Beichuan fault (the central fault of the Longmenshan), and the Anxian–Gongxian fault (the front of the Longmenshan fault). Among them, the Yingxiu–Beichuan rupture was the main seismic rupture of the Wenchuan earthquake, and a surface rupture zone of about 270 km was formed along this rupture zone in and around the epicenter area, which was the longest surface rupture zone in the Wenchuan earthquake. The distribution and activity characteristics of these rupture zones provide a key reference for the causes of the earthquake and the tectonic environment of the epicenter area.

The maximum value of the 0–5 Hz spectral amplitude envelope of the continuous seismic waveform data of each hourly segment is taken as the anomalous signal tracking object, which is denoted as MASE (Maximum Amplitude of Spectrum Envelope) here. Figure 2 shows the location distribution of fixed seismic stations around the epicenter of the Wenchuan earthquake, with special labels for the six key stations used in this study (JYA, JMG, AXI, DFU, JJS, and YZP). These stations are all located at different positions around the epicenter and the Longmenshan Fracture Zone, covering seismic wave data from the epicenter area and the surrounding regions. Through data acquisition at these stations, the seismic waveform records in and around the epicenter area can be obtained, thus capturing dynamic changes in seismic activities comprehensively in time and space. The distribution of these stations provides sufficient data support and geographic coverage for studying the extraction of pre-seismic low-frequency signals, the analysis of acoustic emission b-values, and the calculation of fractal dimensions, ensuring the representativeness and accuracy of the data acquisition.

### 3.2. Marginal Spectrum

Marginal spectra are commonly used to characterize the frequency distribution of a single variable or multiple variables in a given system [13]. In seismology, marginal spectrum analysis is a key time–frequency analysis method in seismic data processing that captures the distribution characteristics of seismic signals at different times and frequencies. This analysis method is of great significance in revealing the complex patterns of seismic signals, their source properties, and energy release processes. Prior to marginal spectral analysis, the seismic data need to be preprocessed to ensure the accuracy of the signal and reduce the noise interference. Common preprocessing steps include filtering, normalization, and segmentation. Filtering is used to remove the noise in the non-target frequency bands to make the main frequency components of the seismic signal clearer; normalization ensures that the amplitude of the signal is consistent in different time segments to avoid the masking of information due to amplitude differences; and segmentation facilitates focusing on the key time windows of the earthquake and improves the efficiency of the time–frequency analysis.

The marginal spectrum is calculated as follows:

The preprocessed signal is first subjected to a Fast Fourier Transform (FFT), which converts the time domain signal into a frequency domain signal:

(15) $$X\left(f\right)=FFT(X(t))$$

The power spectral density at each frequency is then calculated from the FFT results:

(16) $$P\left(f\right)=\frac{1}{N}{\left|X(f)\right|}^{2}$$

where N is the signal length.

If there are multiple channels of data, calculate the average of the power spectra for each frequency to obtain the marginal spectra:

(17) $$P_{marginal}\left(f\right)=\frac{1}{M}\sum_{i=1}^{M}P_{i}(f)$$

where M is the number of channels, and P_i_(f) is the power spectrum of the ith channel.

Marginal spectrum analysis has a unique advantage in seismic data processing, providing data support for in-depth research in the fields of earthquake source mechanisms and earthquake engineering by revealing the time–frequency structure of seismic signals. This method not only helps researchers to understand the physical mechanism of earthquake occurrence more accurately but also provides a scientific basis for subsequent earthquake early warning and earthquake prevention and mitigation work.

### 3.3. Calculation of Acoustic Emission b-Value and Information Entropy

**Figure 3:**
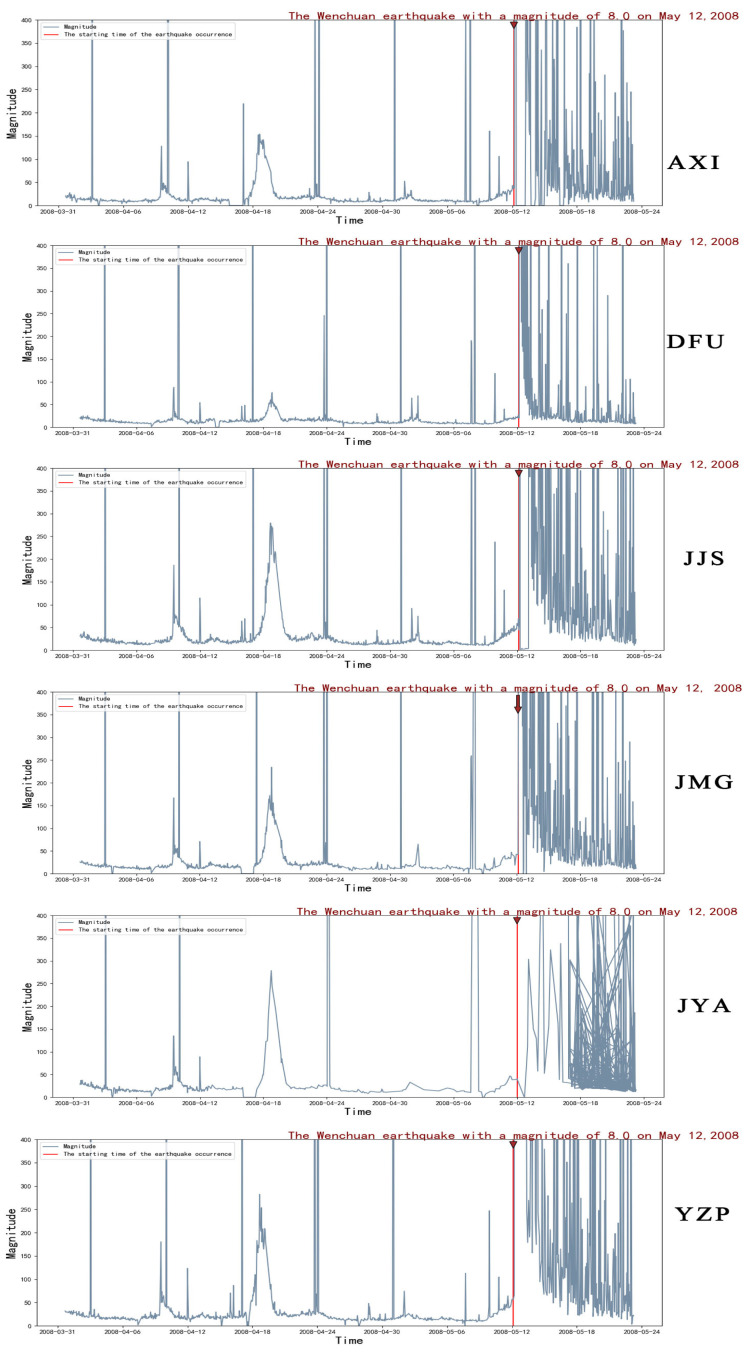
Time course of the maximum magnitude of the marginal spectra at stations AXI, DFU, JJS, JMG, JYA, and YZP.

Continuous seismic waveform data from six seismic stations (JYA, JMG, AXI, DFU, JJS, and YZP) near the 2008 Wenchuan 8.0 earthquake were selected for analysis. The data recorded at each hour were spectrally analyzed to obtain the maximum magnitude of the marginal spectrum for that period. Then, the marginal spectral maximum magnitude of the same station at different hourly segments was continuously tracked, and the results obtained are shown in Figure 3.

The collected data should be standardized. For each station, a sliding window containing six consecutive acoustic emission events was applied to compute the b-value (Equation (8)) and Shannon entropy (Equation (14)). The window length of the six events was empirically selected to balance temporal resolution and statistical robustness, ensuring a sufficient data density for reliable parameter estimation while capturing short-term precursory variations. A step size of one event was adopted for the sliding window, allowing an overlapping analysis to enhance the temporal resolution while maintaining computational efficiency. Place the sliding window at the start of the data sequence and determine the initial data within the window. Detect acoustic emission events within the data segment in the window, which is typically based on characteristics such as the amplitude and duration of the signals. Count the number of acoustic emission events detected within the window and calculate the total or average energy of the acoustic emission signals within the window. This is usually achieved by squaring, summing, or integrating the signal amplitudes. Then calculate the acoustic emission b-value and the value of information entropy for the data in each window. Finally, slide the window forward according to the set step size to ensure that new data points are included in the window range and old data points are removed. Repeat the steps of event detection, energy calculation, and b-value calculation until the window covers the entire data sequence.

It should be noted that the calculation method and interpretation of the acoustic emission b-value may vary depending on the specific application area and material type. Therefore, when performing an acoustic emission analysis, it is necessary to select the appropriate calculation method and interpretation criteria according to the actual situation.

### 3.4. Analysis of b-Value and Information Entropy Calculation Results

**Figure 4:**
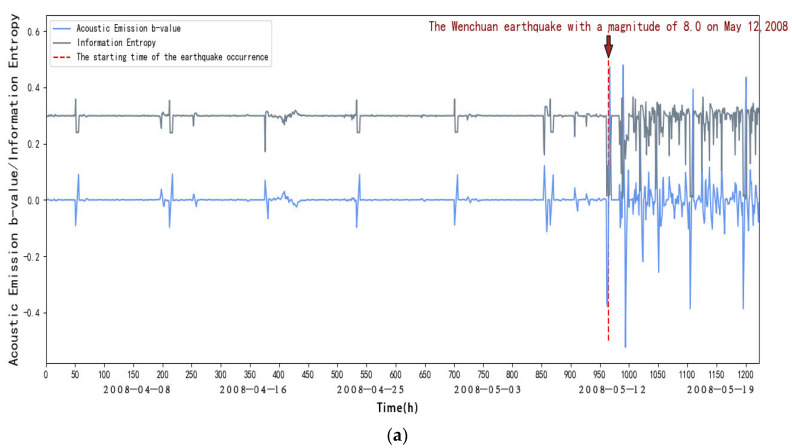

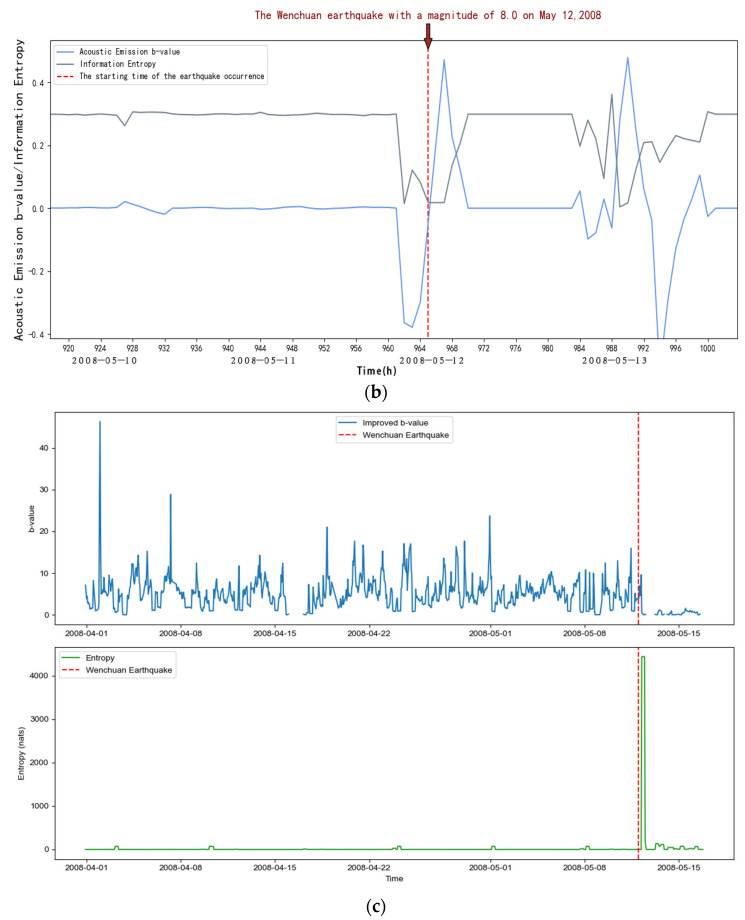
(**a**) Variation in acoustic emission b-value and information entropy with time at AXI station. (**b**) Changes in acoustic emission b-values and information entropy over time at AXI station (amplification). (**c**) New b-values and information entropy over time at AXI station.

The acoustic emission b-value and information entropy of the JYA, JMG, AXI, DFU, JJS, and YZP stations can be calculated by the above steps, as shown in Figure 4:

From Figure 4, we can see that within the first 40 days (from 1 April to 10 May 2008), in the elastic phase, the frequency of small acoustic emission events increases, and the energy release increases when loading to the peak due to the Kaiser effect, the b-value decreases, and the entropy of the information remains stable. After 40 days (after the 10 May 2008), it is in the inelastic stage, the frequency of acoustic emission large events increases sharply, the energy release is intensified, and with the increase in stress, characterized by the rapid decrease in the rate of acoustic emission small events and the beginning of the increase in large events, at this time the b-value will fall off a cliff, and at the same time, there will be an obvious decrease in information entropy. This indicates that abnormal seismic activity will occur, and it is in the critical stage.

**Figure 5:**
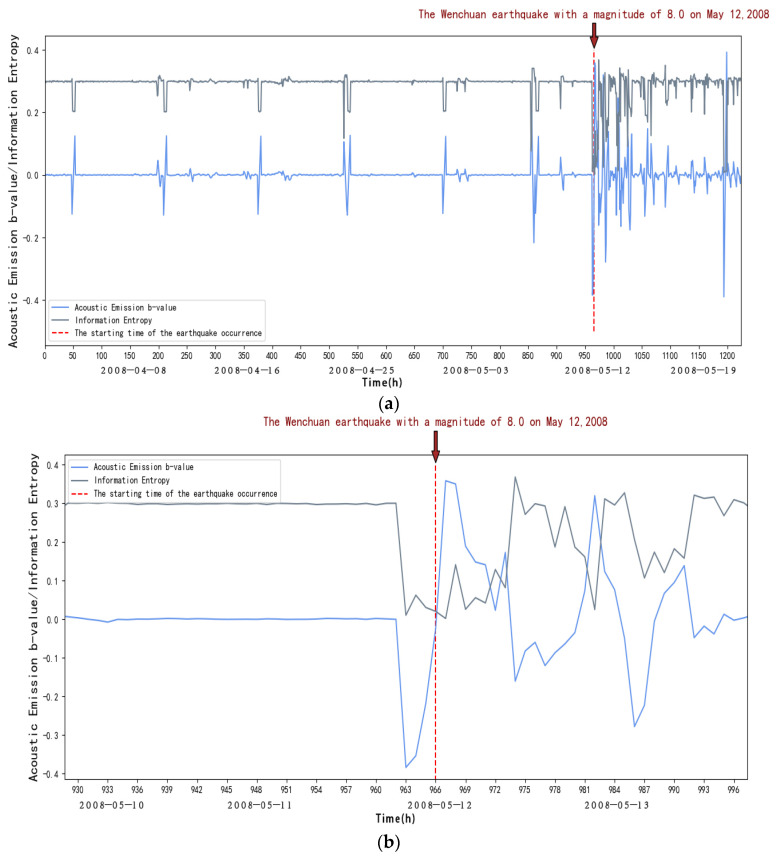

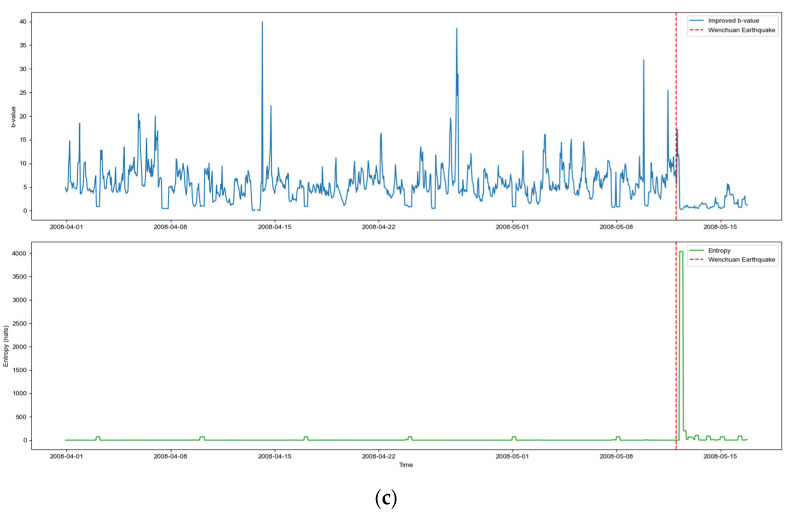
(**a**) Variation in acoustic emission b-values and information entropy over time at DFU station. (**b**) Changes in acoustic emission b-values and information entropy over time at DFU station (amplification). (**c**) New b-values and information entropy over time at DFU station.

From Figure 5, we can see that within the first 40 days (1 April to 11 May 2008), in the elastic phase, the frequency of acoustic emission small events increases, and the energy release increases, when loading to the peak, due to the Kaiser effect; at this time the b-value is relatively small, and at this time the information entropy does not change significantly; after 40 days (after 11 May 2008), in the nonelasticity stage, the frequency of acoustic emission large events increases sharply, the energy release is intensified with the increase in stress, characterized by a rapid decrease in the rate of acoustic emission small events, and large events began to increase; at this time, the b-value will appear to fall off a cliff, and the information entropy begins to fall; there is a clear process of entropy reduction. This indicates that seismic activity anomalies will occur, which are at the critical stage.

**Figure 6:**
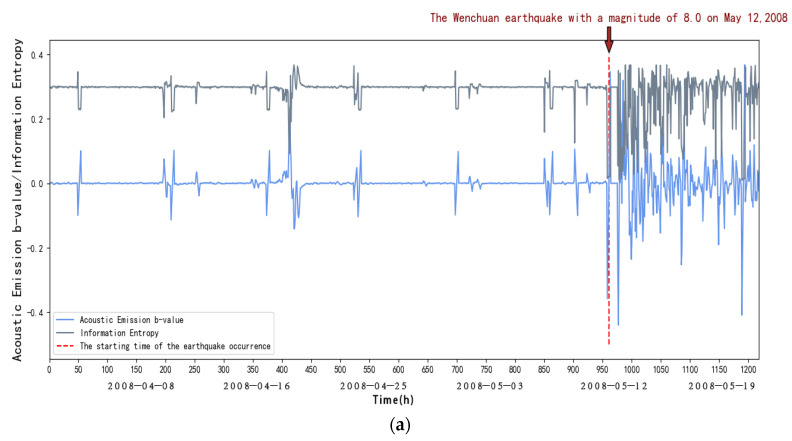

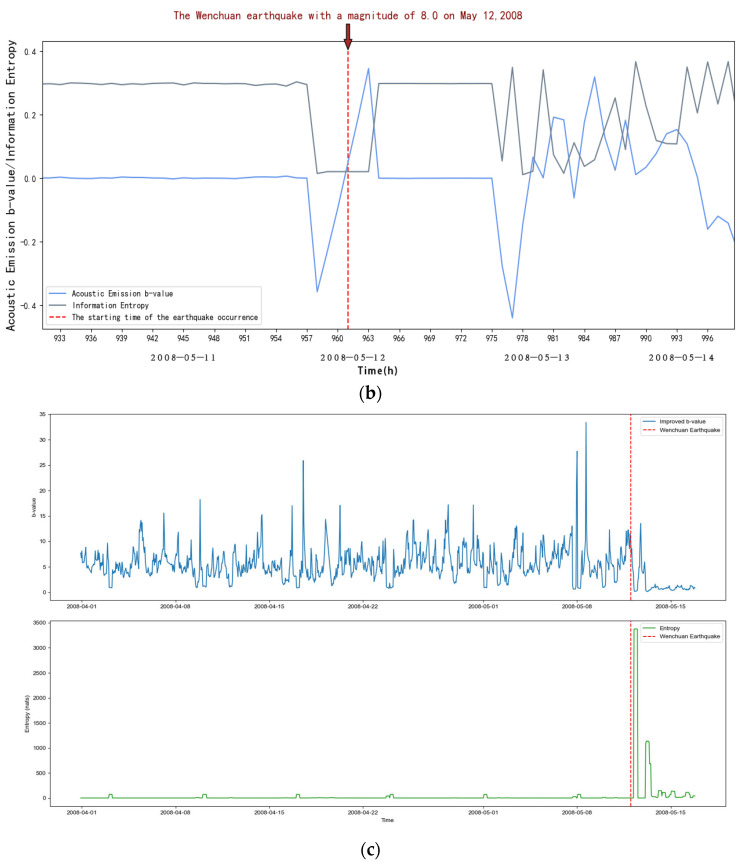
(**a**) Variation in acoustic emission b-value and information entropy with time at JJS station. (**b**) Changes in acoustic emission b-value and information entropy over time at JJS station (amplification). (**c**) New b-values and information entropy over time at JJS station.

From Figure 6, we can see that within about the first 39 days (from 1 April to 11 May 2008), it is in the elastic phase, the frequency of the acoustic emission small events increases, the energy release increases with it, and when loading reaches the peak, the b-value decreases relatively at this time due to the Kaiser effect, and the information entropy at this time does not change significantly; after about 39 days (after 11 May 2008), it is in the inelastic phase, the frequency of acoustic emission large events increases sharply, the energy release is intensified, and with the increase in stress, characterized by the rapid decrease in the rate of acoustic emission small events and the beginning of the increase in large events, at this time the b-value will appear to fall off a cliff, and at the same time, there is an obvious decreasing entropy process for the information entropy. This indicates that abnormal seismic activity will occur, and it is in the critical stage.

**Figure 7:**
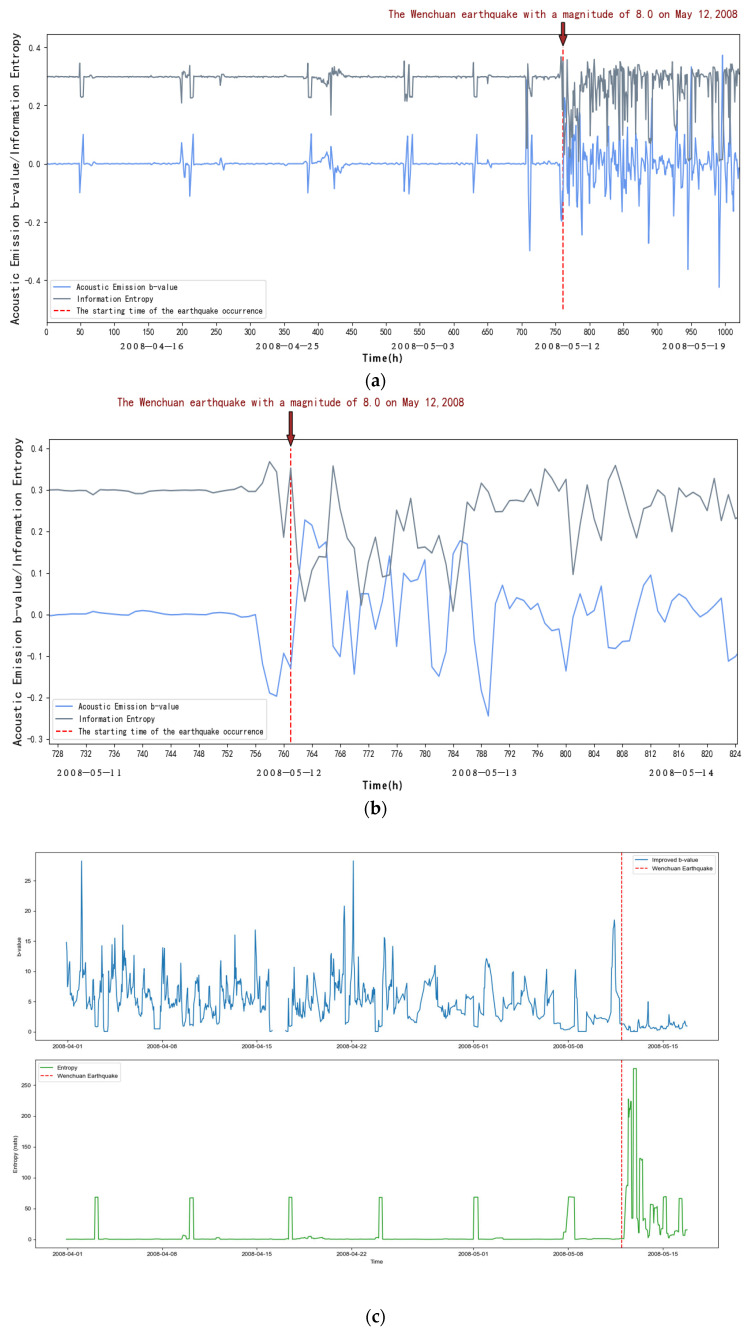
(**a**) Variation in acoustic emission b-value and information entropy with time at JMG station. (**b**) Changes in acoustic emission b-value and information entropy over time at JMG station (amplification). (**c**) New b-values and information entropy over time at JMG station.

From Figure 7, we can see that within about the first 31 days (1 April to 1 May 2008), in the elastic phase, the frequency of small acoustic emission events increases, and the energy release increases when loaded to the peak, due to the Kaiser effect; at this time the b-value decreases relatively, and the entropy of the information does not change significantly; after about 31 days (after 1 May 2008), in the inelastic stage, the frequency of acoustic emission large events increases sharply, the energy release is intensified with the increase in stress, characterized by a rapid decrease in the rate of acoustic emission small events, and large events began to increase; at this time, the b-value will appear to fall off a cliff, and at this time, the information entropy also appears to be a reduced phenomenon. This indicates that abnormal seismic activity will occur, and it is at the critical stage.

**Figure 8:**
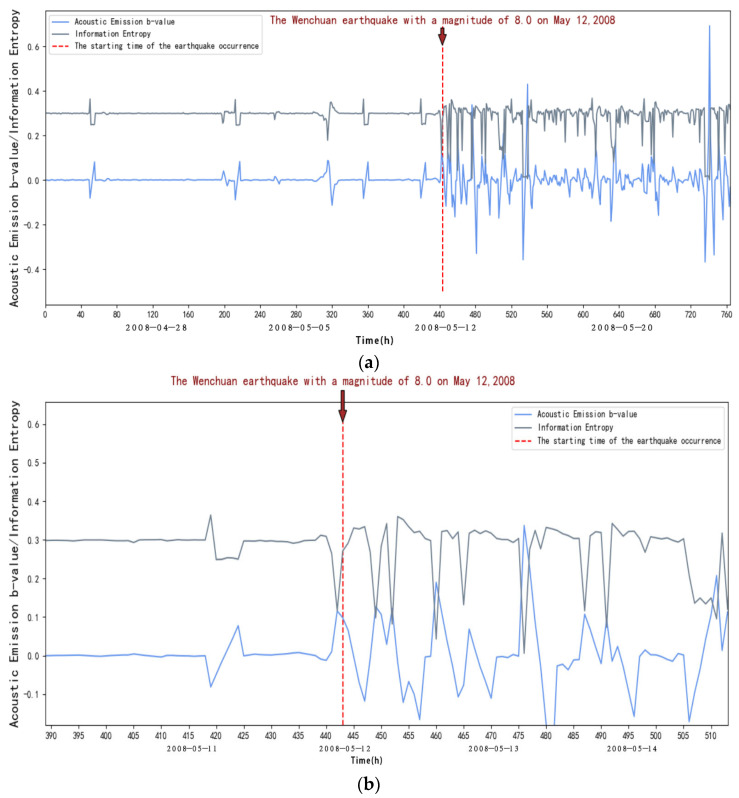

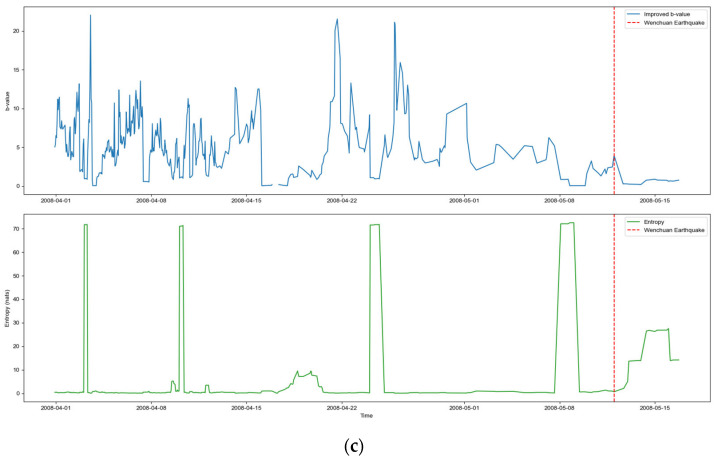
(**a**) Variation in acoustic emission b-value and information entropy with time at JYA station. (**b**) Changes in acoustic emission b-value and information entropy over time at JYA station (amplification). (**c**) New b-values and information entropy over time at JYA station.

From Figure 8, we can see that within about the first 17 days (1 April to 19 April 2008), in the elastic phase, the frequency of small acoustic emission events increases, and the energy release increases when loading to the peak due to the Kaiser effect; at this time, the b-value decreases, and at this time, the entropy of the information is kept stable. After 17 days (after 4 April 2008), it is in the inelastic phase, the frequency of large acoustic emission events increases sharply, and the energy release is intensified with the increase in stress, characterized by a rapid decrease in the rate of small acoustic emission events and the beginning of an increase in the number of large events; at this time, the b-value will appear to fall off a cliff, and at this time, the information entropy is decreasing, which indicates that the order of the seismic activity is increasing, and the disorder is reducing. This indicates that seismic activity anomalies will occur, which are at the critical stage.

**Figure 9:**
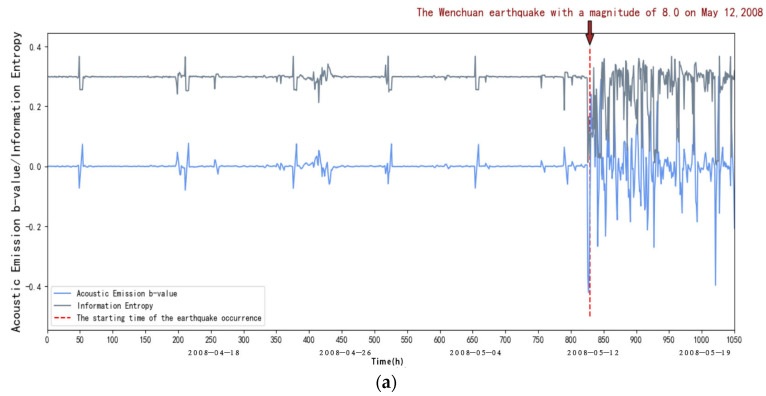

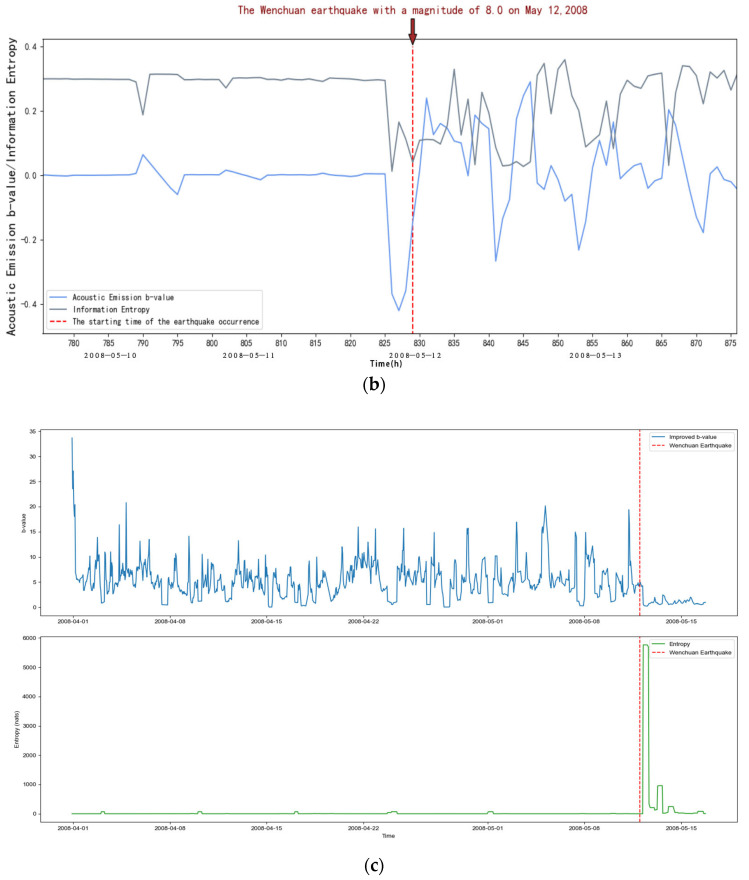
(**a**) Variation in acoustic emission b-value and information entropy with time at YZP station. (**b**) Changes in acoustic emission b-value and information entropy over time at YZP station (amplification). (**c**) New b-values and information entropy over time at YZP station.

From Figure 9, we can see that in about the first 34 days (1 April to 4 May 2008), in the elastic phase, the frequency of acoustic emission small events increases, and the energy release increases when loading to the peak due to the Kaiser effect; at this time, the b-value decreases, and at this time, the entropy of the information is kept stable. After 34 days (after 4 May 2008), in the inelastic phase, the frequency of large acoustic emission events increases sharply, and the energy release is intensified with the increase in stress, characterized by a rapid decrease in the rate of small acoustic emission events and the beginning of an increase in large events; at this time, the b-value will appear to fall off a cliff, and at this time, the entropy of the information will have a significant decrease. This indicates that anomalous seismic activity will occur, and it is in the critical stage.

To summarize, we can find that the information entropy will go through a process of decreasing entropy before the occurrence of strong earthquakes, and at the same time, the acoustic emission b-value will drop suddenly, and it can be seen through observation that the acoustic emission b-value will increase until it reaches the peak after a sudden drop, and a strong earthquake generally occurs before and after the peak; according to this, we can take the acoustic emission b-value and the sudden decrease in information entropy as a basis for the identification of strong earthquakes and predicting the time of the earthquake according to the acoustic emission b-value. We can also predict the time of earthquake occurrences based on the b-value of acoustic emission.

As shown in Figure 4, Figure 5, Figure 6, Figure 7, Figure 8 and Figure 9, to address the deviations in traditional b-value calculations caused by small sample sizes and incomplete earthquake catalogs, this study introduces the dynamic adjustment of the completeness magnitude within moving windows. This approach enhances sensitivity to pre-seismic stress variations by applying weighted corrections to mitigate the impact of data omissions. For information entropy computation, we implement kernel density estimation (KDE) to replace conventional binning statistics, eliminating entropy interference from arbitrary bin boundaries through a continuous probability density estimation. An integrated anomaly detection and processing mechanism effectively resolves numerical instability issues arising from data sparsity or homogeneity. This refined methodology demonstrates enhanced noise resistance in the Wenchuan earthquake case study, successfully capturing the coordinated evolution characteristics of pre-seismic b-value decline and entropy reduction with improved clarity. These advancements provide more reliable criteria for identifying seismic critical states [14].

The proposed dynamic completeness magnitude adjustment within moving windows significantly improves the accuracy of b-value estimation by adaptively compensating for catalog incompleteness and small-sample biases, while weighted corrections enhance the sensitivity to localized stress variations [3]. This method demonstrates robust noise resistance in the Wenchuan earthquake case study, capturing precursory b-value declines with higher resolution. However, its computational complexity increases with window size optimization and real-time adjustments, potentially limiting the rapid processing of large datasets. Additionally, the dependency on high-quality instrumentation records and regional seismicity patterns necessitates careful parameter calibration to avoid overfitting in diverse tectonic environments.

## 4. Conclusions and Exploration

### 4.1. Conclusions

In this study, we conduct a retrospective study of the acoustic emission b-value and information entropy of the acoustic emission before the 2008 Wenchuan magnitude 8.0 earthquake in an earthquake example to reveal the sub-stabilization stage of the rock mass, the orderly change in the rupture of the fissure, and the whole process of subterranean structural destabilization. These changes provide an important theoretical basis and practical guidance for our understanding of earthquake precursors. Based on these changes, we can draw the following conclusions:

(1) From 1 April to 10 May 2008, the b-value and information entropy were relatively stable, indicating that the rock mass was in the sub-stability stage, and the acoustic emission events and energy gradually increased, reflecting that the stress was accumulating and had not yet led to destruction. This stage is characterized by the accumulation of stress in the rock body, which has not yet reached the critical point of destruction.

(2) After 10 May 2008, the decrease in b-value and the decrease in information entropy indicate that the rift of the rock body had shifted from disorder to order, and the possibility of seismic activity had increased. The sudden decrease in the b-value reflects the fact that the stress inside the rock body had reached the critical point, and the rupture phenomenon had begun to appear in a concentrated manner. At this time, the decrease in information entropy reflected the increase in the orderliness of the system, which predicted a significant increase in the possibility of seismic activity.

(3) From 11 May to 12 May 2008, the acoustic emission b-value and information entropy decreased significantly, indicating that it was in the critical stage of the earthquake, when the crustal stress reached its limit, and then the b-value rebounded to the peak, reflecting the fragility of the rock mass. During the critical stage before the strong earthquake, the whole underground structure was gradually destabilized. During this period, the sharp decrease in the acoustic emission b-value and the significant decrease in the information entropy together indicated that the crustal stress had reached the limit state. The acoustic emission b-value underwent a rebound process again and finally reached a peak value before the strong earthquake. This phenomenon not only indicates the fragility of the rock mass but also provides an important reference for earthquake prediction.

In summary, by systematically analyzing the dynamic changes in the acoustic emission b-value and information entropy, we can identify the key time points of rock mass sub-stability, the orderly transition of rift rupture, and the subsurface structural destabilization. These findings provide strong support for research into subsequent seismic event prediction. Additionally, we emphasize the potential of combining acoustic emission technology with information entropy theory. Through the integration of these two important indexes, researchers can more comprehensively capture the changing trends in seismic activity, thus improving the timeliness and accuracy of early warning.

### 4.2. Exploration

The combination of acoustic emission b-value and information entropy provides new ideas and methods for China’s earthquake-monitoring and early warning system. By integrating the changing characteristics of the acoustic emission b-value and information entropy, researchers can more accurately identify potential seismic activities and develop effective prediction strategies. This combined research method provides new perspectives for earthquake science and may play an important role in future earthquake prediction practice. Although the changes in b-values and information entropy indicate potential precursor significance, earthquake prediction still requires the joint analysis of multiple parameters and long-term validation. The results of this study provide a theoretical reference for the design of physical mechanism-driven early warning systems, rather than a deterministic predictive tool. While the dual-parameter framework shows potential for improving earthquake forecasting, operational implementation faces significant challenges: (1) Sparse station coverage limits the spatial resolution of precursor signals, particularly in regions with complex fault systems. (2) Ambient noise (e.g., anthropogenic activities or non-tectonic tremors) may distort b-value estimates, necessitating advanced filtering techniques such as wavelet denoising. (3) Real-time data transmission and processing remain bottlenecks for operational systems, requiring a scalable cloud computing infrastructure. Addressing these challenges requires multi-disciplinary collaboration, including improved sensor deployment, machine learning-based noise suppression, and cloud computing infrastructure. These limitations highlight that earthquake prediction remains a probabilistic endeavor, dependent on the continuous refinement of monitoring networks and analytical frameworks.

This retrospective analysis of the dual-parameter framework provides a theoretical foundation for enhancing earthquake precursor detection in the Wenchuan region. Our results suggest that coordinated declines in b-value and entropy may serve as indicators of a critical stress accumulation, although their operational utility requires validation in diverse tectonic settings. It is critical to note that this study is a post-event analysis of the 2008 Wenchuan earthquake. The geological complexity of the Longmenshan Fault Zone may limit the direct extrapolation of these findings to other regions. Future work must test the generalizability of the b-value–entropy synergy in varying tectonic regimes, such as strike-slip faults or subduction zones, to assess its universal applicability. Meanwhile, combining advanced machine learning technology to analyze and update the prediction model in real time will make earthquake prediction more prospective and accurate, thus better protecting people’s lives and properties.

## Data Availability

The data are sourced from the “China Seismic Array Data Center” of the Institute of Geophysics, China Earthquake Administration, and the Earthquake Administration of Sichuan Province.
